# Epitope-level analysis of cross-reactive human HLA antibodies against genetically modified swine leukocyte antigens in xenotransplantation

**DOI:** 10.3389/fimmu.2025.1712793

**Published:** 2025-12-15

**Authors:** Sang-Ik Cho, Ji-Jing Yan, Beom Seok Kim, Nayoung Ko, Joohyun Shim, Hyunil Kim, Eun-Jee Oh

**Affiliations:** 1 Department of Medical Sciences, Graduate School of the Catholic University of Korea, Seoul, Republic of Korea; 2 The Research Institute for Transplantation, Yonsei University College of Medicine, Seoul, Republic of Korea; 3 Division of Nephrology, Department of Internal Medicine, Yonsei University College of Medicine, Seoul, Republic of Korea; 4 Department of Transgenic Animal Research, Optipharm Inc., Cheongju, Republic of Korea; 5 Department of Laboratory Medicine, Seoul St. Mary’s Hospital, College of Medicine, The Catholic University of Korea, Seoul, Republic of Korea; 6 Research and Development Institute for In Vitro Diagnostic Medical Devices, College of Medicine, The Catholic University of Korea, Seoul, Republic of Korea

**Keywords:** xenotransplantation, eplet, cross-reactivity, human serum, antibody elution

## Abstract

**Introduction:**

Xenotransplantation using genetically modified pigs is a promising solution to organ shortages, particularly for highly sensitized patients with broad anti-HLA sensitization who lack compatible allografts. However, preformed human anti-HLA antibodies may cross-react with porcine SLA, posing a barrier for clinical application. This study aims to characterize the extent and specificity of cross-reactive antibody responses against genetically engineered pig PBMCs, particularly from quadruple knockout (QKO) pigs.

**Methods:**

We evaluated antibody binding and cytotoxicity of 68 human sera stratified by HLA class I and II antibody profiles using flow cytometric crossmatch (FCXM), complement-dependent cytotoxicity (CDC) assays (CDC-NIH and CDC-AHG), antibody elution, and single antigen bead assays. Porcine PBMCs from wild-type and gene-edited pigs (GTKO, TKO, QKO) were used. High-resolution SLA epitope mapping was performed with antibody eluted from select sera followed by in silico sequence and structural analyses.

**Results:**

Human sera showed strong IgG and IgM binding to wild-type pig PBMCs, which was significantly reduced by RBC adsorption, whereas binding to QKO pig PBMCs lacking key glycan xenoantigens was minimal and unaffected by RBC adsorption. Sensitized human sera with both HLA class I and II antibodies demonstrated significantly elevated IgG binding to QKO pig PBMC T and B cells compared to antibody-negative sera (p < 0.05). CDC-AHG assays revealed increased cytotoxicity titers (≥1:8) in HLA antibody-positive sera versus negatives (p < 0.01). Antibody elution from five crossmatch-positive sera identified predominant class I eplets (62EE, 162GLS, 163LG, 163LS/G, 166ES, 199V) and class II DR epitopes (13SE, 37F, 47F, 70D, 70DA) that target SLA 4.5/6.7 haplotypes. In contrast, anti-HLA-DQ and -DP reactivity was limited post-elution. Structural modeling confirmed that these epitopes are conserved and surface-exposed in SLA alleles.

**Conclusion:**

Cross-reactive anti-SLA antibodies are common in highly sensitized human sera, driven by antibody specificity and epitope conservation. Despite glycan xenoantigen deletion, sensitized sera maintain IgG-mediated cross-reactivity and cytotoxicity against gene-edited pig cells. These findings highlight the need for detailed epitope-level analysis to refine immunologic risk assessment and recipient selection to reduce antibody-mediated rejection in clinical xenotransplantation.

## 1 Introduction

Xenotransplantation has emerged as a promising solution to resolve the critical shortage of donor organs for patients with end-stage organ failure. Among various xenogeneic sources, genetically engineered pigs are widely recognized as optimal donors due to their physiological and anatomical similarities to humans, combined with the feasibility of precise genetic modifications (1–3). To overcome immunological barriers, extensive research has focused on eliminating major xenoantigens that trigger hyperacute rejection and early graft failure. The α1,3-galactose (α-Gal) epitope, generated by the GGTA1 gene, was removed in GGTA1-knockout (GTKO) pigs to reduce early xenograft rejection. Subsequent triple (TKO: GGTA1, CMAH, B4GALNT2) and quadruple (QKO: GGTA1, CMAH, B4GALNT2, A3GALT2) knockout pigs further minimized human antibody binding (1, 4, 5). Combined with optimized immunosuppressive regimens, these advances have enabled significant progress in preclinical non-human primate studies (5–8), studies in brain-dead human recipients (9–12), and recent clinical xenotransplantation trials in living patients (13–15).

Despite these advances, a critical concern persists regarding the potential cross-reactivity between preformed anti-human leukocyte antigen (HLA) antibodies and swine leukocyte antigens (SLA). This issue is particularly relevant for highly sensitized patients with broad HLA reactivity, who represent prime candidates for xenotransplantation due to their limited access to compatible human allografts. In previous studies, sera from patients sensitized to HLA class I have demonstrated binding to SLA class I epitopes (16, 17), and antibodies against HLA class II have also reacted with SLA class II antigens (18). Notably, such reactivity persists despite adsorption with porcine red blood cells (RBCs), designed to remove anti-glycan antibodies, suggesting the presence of antibodies targeting SLA or other xenoantigens (19). Moreover, xenoreactive antibody responses have been detected in commonly used human blood products, indicating the prevalence of cross-reactive humoral immunity (20). These findings are supported by preclinical studies in non-human primates, where allosensitization has been associated with accelerated xenograft rejection (21). Collectively, these findings suggest that anti-HLA antibodies may contribute to xenograft rejection through cross-reactivity, emphasizing the importance of careful recipient selection.

Histocompatibility testing plays a critical role for assessing immunologic risk prior to transplantation, enabling risk stratification and prevention of antibody-mediated rejection (22). In allotransplantation, development of advanced assays—including complement-dependent cytotoxicity (CDC), flow cytometric crossmatch (FCXM), and single antigen bead (SAB) assay—has significantly enhanced the detection of HLA-specific antibodies and enabled epitope-level analysis (23). These methodologies, particularly when combined with techniques such as adsorption with crossmatch cells and elution (AXE) protocols, can enhance the sensitivity for detecting clinically relevant cross-reactive antibodies while minimizing background noise (24). However, xenotransplantation lacks standardized crossmatching protocols, and comprehensive studies correlating xenoreactive antibodies with detailed HLA antibody profiles in sensitized individuals remain limited (25).

In this study, we evaluated antibody reactivity in human sera against genetically modified porcine peripheral blood mononuclear cells (PBMCs), stratified by HLA class I and class II sensitization profiles. Using complementary approaches—including FCXM, antibody elution and SAB assay with epitope-level analysis—we sought to characterize the extent and specificity of cross-reactive antibody binding between human anti-HLA responses and porcine xenoantigens.

Our primary objectives were to: (1) assess the relationship between HLA antibody status and xenoreactive antibody binding to gene-edited pig cells, (2) evaluate the impact of RBC adsorption on assay sensitivity and specificity, and (3) identify specific epitopes mediating cross-reactivity. This work aims to provide critical insights into immunological risk assessment for highly sensitized xenotransplant candidates and to inform the development of evidence-based recipient selection strategies for future clinical trials.

## 2 Materials and methods

### 2.1 Human sera

Human sera were obtained from residual specimens submitted for HLA antibody tests from transplant waiting patients at Seoul St. Mary’s Hospital. HLA antibody screening was performed using the LABScreen Single Antigen assays (One Lambda Inc., A Thermo Fisher Scientific brand, Canoga Park, CA, USA), which allowed for the detection and characterization of class I and class II HLA-specific antibodies. A total of 68 human sera were categorized based on their HLA antibody profiles. Of these, 7 samples that showed negative to weak (median fluorescence intensity (MFI) <3,000) reactivity for both HLA class I and class II antigens were used in comparative analyses of antibody binding across porcine PBMCs from different gene edited backgrounds. The remaining 61 sera were categorized as HLA antibody-positive based on MFI cutoff of >10,000, indicating strong allosensitization; 10 positive for both HLA class I and II antibodies, 10 positive for HLA class I only, 10 positive for HLA class II only, and 31 negative for both HLA class I and class II antibodies. Each human serum was used for a single experimental run in flow cytometric crossmatch (across three porcine PBMCs), RBC adsorption, complement-dependent cytotoxicity, elution, and single antigen bead assays, with negative and positive controls included in all procedures for validation. This study was approved by the Institutional Review Board of Seoul St. Mary’s Hospital (KC24SISI0367), with informed consent waived in accordance with institutional guidelines, as only residual biospecimens remaining after routine clinical testing were used.

### 2.2 Porcine cell isolations

Porcine whole blood was obtained from various genetically modified pigs provided by Optipharm Inc. (Cheongju, Korea). The pig genotypes included wild-type (WT) pigs, GTKO pig with knockout of GGTA1 gene, TKO pigs, lacking GGTA1, CMAH, and B4GALNT2, QKO pigs, with TKO plus A3GALT2 knockout, TKO.hCD55.hCD39 pigs expressing human complement regulatory proteins hCD55 and hCD39, TKO.hCD46.hTBM pigs expressing hCD46 and thrombomodulin (4, 26, 27). Among the various genetically modified pigs, QKO pig PBMCs with high-resolution SLA typing were used for cross-reactivity testing and epitope analysis with allosensitized human sera. Porcine PBMCs were isolated from heparinized whole blood using standard density gradient centrifugation with Ficoll-Paque™ PLUS (Cytiva, Marlborough, MA, USA). Briefly, the porcine whole blood was diluted 1:1 with phosphate-buffered saline (PBS), layered over Ficoll, and centrifuged at 2,000 rpm for 30 min. The buffy coat was harvested, washed twice with PBS (2000 rpm for 5 min), and the resulting PBMCs were resuspended in 90% fetal bovine serum (Thermo Fisher Scientific, Waltham, MA) supplemented with 10% dimethyl sulfoxide (Sigma-Aldrich, St. Louis, MO, USA), and stored at -80°C and then transferred to liquid nitrogen (-196°C) for long-term storage. Viability of PBMCs post-thaw was assessed using acridine orange/propidium iodide stain (Logos Biosystems, Anyang, Korea), and only samples with >80% viability was used for subsequent assays. Porcine RBCs were also isolated from heparinized blood. The blood was centrifuged at 500 × g for 5 min, the packed RBC layer was collected, and cells were washed three times with PBS. The isolated RBCs were stored at a 1:2 ratio in Alsever’s solution (Sigma-Aldrich) and used for antibody adsorption protocols.

### 2.3 Flow cytometric crossmatch assay

2.5 × 10^5^ porcine PBMCs were suspended in 25 μL of eBioscience™ Flow Cytometric Staining Buffer (eBioscience, Thermo Fisher Scientific, Waltham, MA, USA) and incubated with 50 μL of each human serum in a 96-well plate for 20 minutes at room temperature (RT). Cells were washed twice with 200 μL PBS at 800 × g for 5 minutes, and stained with ViaDye™ Red Viability Dye (Cytek Biosciences, Fremont, CA, USA) at 4°C for 30 min. After washes, cells were resuspended and stained at 4°C for 30 min with goat anti-human IgG (FITC), goat anti-human IgM (DyLight 405) (Jackson ImmunoResearch, West Grove, PA, USA), mouse anti-porcine CD21 (PE) and mouse anti-porcine CD3ϵ (SPRD) (SouthernBiotech, Birmingham, AL, USA). Following additional washes, samples were acquired and analyzed using a Cytek Northern Lights flow cytometer (Cytek Biosciences). Negative control included PBMCs incubated with staining buffer alone. MFI values were calculated by subtracting the MFI of the negative control from that of each test sample.

### 2.4 Red blood cell adsorption assay

Porcine RBCs stored in Alsever’s solution (Sigma-Aldrich) were centrifuged at 1,500 × g for 5 minutes to remove the preservative. RBCs were washed three times with PBS at 500 × g for 5 minutes each and incubated with human serum at a ratio of 1:10 at 4°C for 60 minutes. Following incubation, samples were centrifuged at 13,000 rpm for 10 minutes, and the supernatant sera were collected in clean microtubes for subsequent FCXM assays.

### 2.5 Complement-dependent cytotoxicity assay

The CDC assay was performed using porcine PBMCs and human sera. For both CDC-NIH and CDC-AHG, 3 × 10^3^ PBMCs in 1 μL were incubated with 1 μL of human sera. In the CDC-NIH assay, samples were incubated at RT for 60 minutes, followed by the addition of 5 μL of rabbit complement (One Lambda, Canoga Park, CA, USA) and a further incubation for 90 minutes. For CDC-AHG, samples were incubated at 37°C for 60 minutes, then washed and incubated with 5 μL of 1% anti-human globulin (AHG) solution and 5 μL of rabbit complement at 37°C for 90 minutes. Cytotoxicity was assessed by acridine orange/ethidium bromide staining (One Lambda) under immunofluorescence microscope. Human sera were tested at six serial dilutions (1:1, 1:2, 1:4, 1:8, 1:16, 1:32). RPMI 1640 with 5% FBS was used as a negative control, and CDC titer was defined as the lowest serum dilution that resulted in cytotoxicity ≥40%.

### 2.6 Antibody elution and single antigen bead assay

To evaluate potential cross-reactivity, antibodies bound to porcine PBMCs were eluted from five allosensitized human sera and then tested using a SAB HLA Luminex assay. Briefly, 3× 10^6^ porcine PBMCs with 70 μL human serum were incubated in a 96-well plate at 37°C with 5% CO_2_ for 30 minutes. Cells were then washed five times with PBS; each wash involved the addition of 200 μL PBS, centrifugation at 800 × g for 1 minute, and removal of unbound antibodies. After the final wash, cells were treated with 50 μL MagSort elution buffer (One Lambda) at RT for 1 minute. The eluates were collected by centrifugation and immediately neutralized with 4 μL of MagSort Neutralization Buffer (One Lambda). Eluted antibodies were analyzed using the LABScreen Single Antigen Bead Assay (One Lambda) according to the manufacturer’s instructions. Briefly, 20 μL of EDTA-treated eluate was incubated with microbeads coated with individual HLA antigens, and bound antibodies were detected using a PE-conjugated anti-human IgG secondary antibody. Fluorescence intensity was measured with the LABScan3D analyzer, and HLA antibody specificity and strength were determined based on MFI. All elution assays were performed in duplicate. The supernatant from the final wash was used as a negative control. To account for the inherently lower background signal in eluate samples compared to whole sera, a positive antibody reactivity threshold was defined as MFI ≥ 100.

### 2.7 In silico analysis of potential cross-reactive HLA eplets and SLA structures

To analyze antibody binding pattern and potential epitope-level cross-reactivity, initial HLA epitope profiles were generated using HLA Fusion 4.7 software (One Lambda Inc.). Identified HLA epitopes were cross-referenced and validated at the allele level through the HLA Eplet Registry (https://epregistry.com.br/; accessed August 12, 2025). To evaluate similarities with porcine MHC, SLA and HLA protein sequences were retrieved from the IPD-MHC database (https://www.ebi.ac.uk/ipd/mhc/; accessed August 5, 2025). SLA sequences were aligned to HLA reference sequences using BLASTP (NCBI, https://blast.ncbi.nlm.nih.gov/; accessed August 5, 2025) to assess positional correspondence. When available, three-dimensional structural data for SLA molecules- including experimentally determined and those predicted by AlphaFold and ESMFold—were obtained from the RCSB Protein Data Bank (https://www.rcsb.org/; accessed August 12, 2025), UniProt (https://www.uniprot.org/; accessed August 12, 2025) and the IPD-MHC database. Structural visualization and analysis were performed using PyMOL (open-source version 3.1.0; PyMOL Molecular Graphics System, Schrödinger, LLC; accessed September 3, 2025).

### 2.8 Statistical analysis

All statistical analyses were performed using Prism 10 (GraphPad Software, San Diego, CA). For repeated measures across matched samples, the Friedman test was applied. Paired two-group comparisons were analyzed using the Wilcoxon matched-pairs signed rank test. For comparisons between independent groups, the Kruskal–Wallis non-parametric one-way ANOVA was applied. Data are presented with test statistics and corresponding p-values. Statistical significance was defined as follows: *p < 0.05, **p < 0.01, ***p < 0.001.

## 3 Results

### 3.1 Comparison of IgG and IgM reactivity to wild-type and gene-edited pig PBMCs in HLA antibody-negative human sera

**Figure 1:**
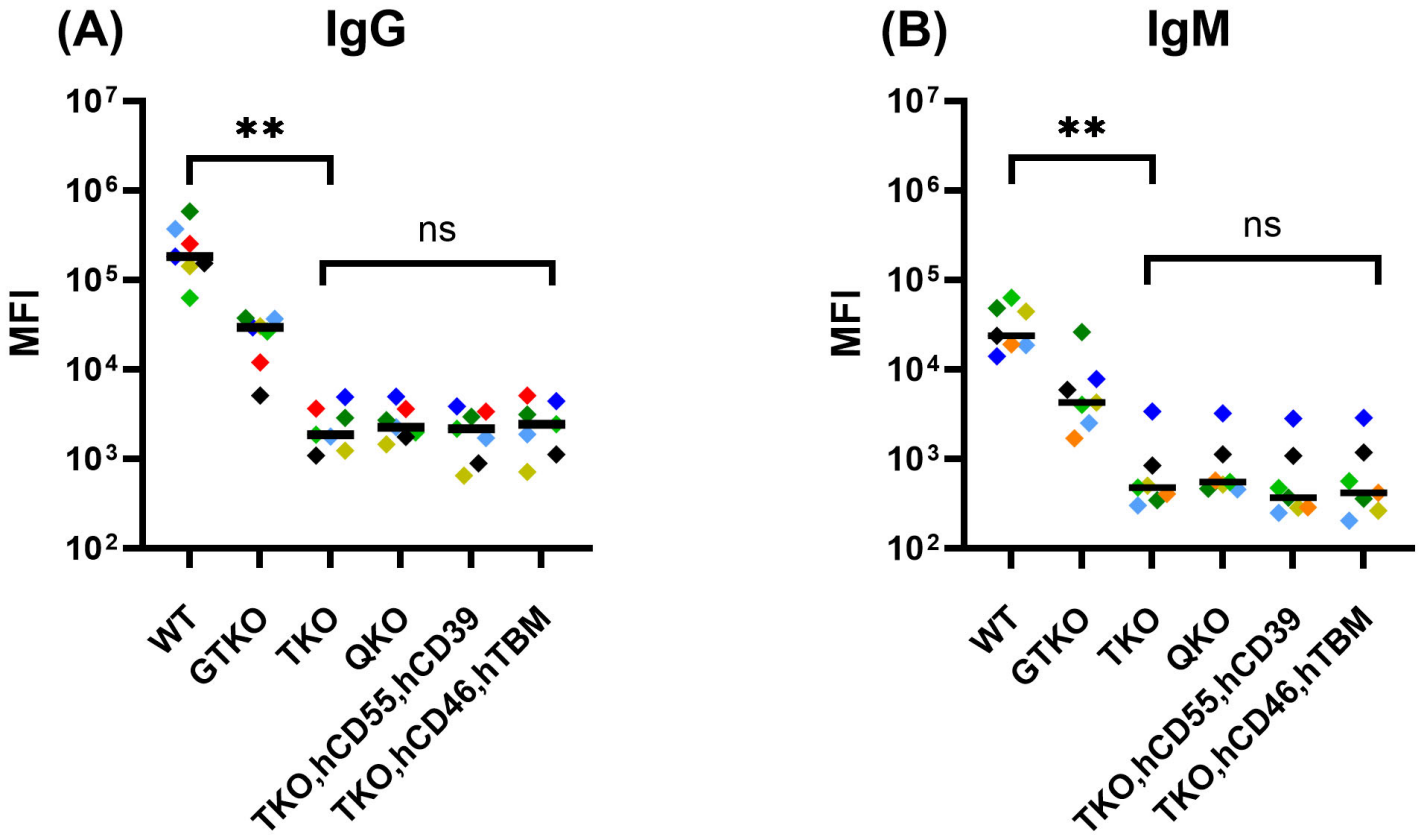
IgG and IgM reactivity of human sera in the absence of strong HLA antibodies against porcine PBMCs from wild-type (WT) and genetically modified pigs. Flow cytometric crossmatch assays were performed to evaluate IgG **(A)** and IgM **(B)** antibody reactivity of seven human sera that were negative or showed only weak reactivity to HLA antibodies against PBMCs from WT and various genetically modified pig lines. WT and GTKO pigs elicited strong antibody responses with high MFI values for both IgG and IgM. In contrast, other genetically modified pigs exhibited significantly reduced MFI values for both IgG (p < 0.01) and IgM (p < 0.01) compared to WT. Each symbol represents a unique human serum sample. **p < 0.01.

To specifically evaluate baseline xenoreactive antibody responses in the absence of anti-HLA sensitization, we performed FCXM assays using seven human sera samples that were negative or showed only weak reactivity for both HLA class I and class II antigens (MFI < 3,000). All sera showed strong IgG and IgM binding to WT and GTKO porcine PBMCs (**Figure 1**). In contrast, IgG and IgM binding to porcine PBMCs with triple carbohydrate deletion (GGTA1/CMAH/B4GALNT2) including TKO, QKO, TKO.hCD55.hCD39, and TKO.hCD46.hTBM showed 25.8 to 219.6-fold and 4.1 to 168.2-fold reductions in MFI values compared with WT for IgG and IgM, respectively. Among these TKO, QKO, and the two other TKO groups, no statistically significant differences were observer (p > 0.05).

### 3.2 Impact of RBC adsorption on IgG and IgM binding to WT and QKO pig PBMCs in human sera

**Figure 2:**
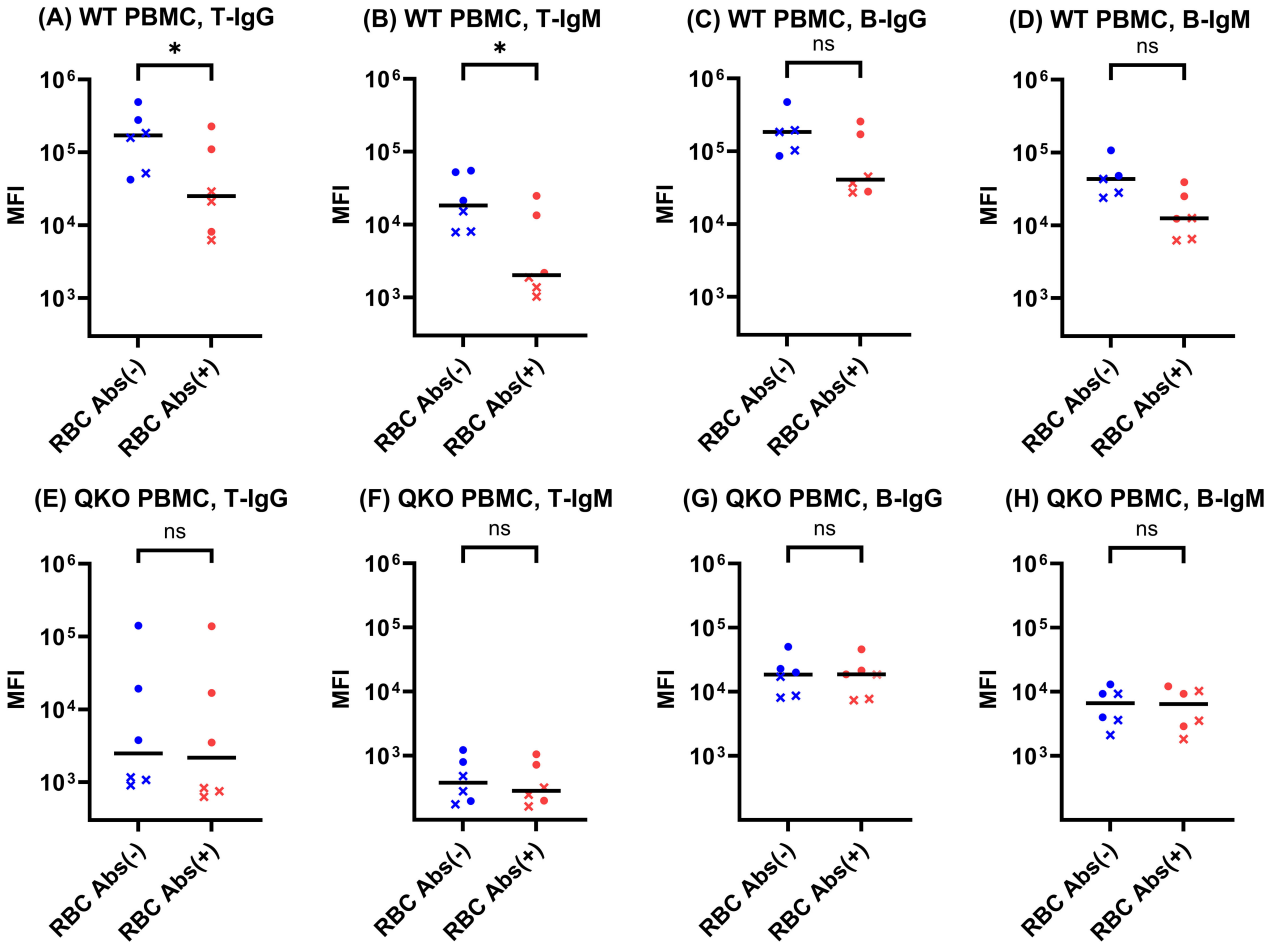
Effect of RBC absorption on IgG and IgM binding to WT and QKO pig PBMCs in six human sera. Flow cytometric crossmatch assays were performed using six human sera (three HLA antibody–positive (●) and three HLA antibody–negative (**×**)) to compare IgG and IgM binding to WT and QKO pig PBMCs, before and after RBC adsorption. RBC adsorption significantly reduced T-IgG and IgM binding to WT PBMCs **(A, B)**, but had no effect on QKO PBMCs **(E–H)**. Each point represents an individual serum; horizontal bars indicate group medians. *p < 0.05; ns, not significant.

To evaluate whether anti-glycan antibodies contribute to IgG and IgM binding to porcine T and B cells, we performed FCXM assays using six human sera—three positive and three negatives for HLA antibodies. Each serum sample was tested against PBMCs from both WT and QKO pigs, with and without prior RBC adsorption. Following RBC adsorption, IgG and IgM binding to WT pig T cells was significantly reduced (both p < 0.05), with a similar trend observed for B cells (both p = 0.063) (**Figures 2A–D**). In contrast, for QKO pig PBMCs, RBC adsorption had no significant effect on either T- or B-cell IgG and IgM reactivity across all tested sera (**Figures 2E–H**). Representative flow cytometric plots of the RBC adsorption are provided in **Supplementary Figure 1**. This result demonstrates that, in the context of QKO pigs lacking major glycan xenoantigens, pre-adsorption with RBCs is unnecessary for FCXM analysis using QKO pig PBMCs, as glycan-mediated background antibody binding is minimal or absent.

### 3.3 IgG and IgM reactivity of human sera to QKO pig PBMCs based on HLA antibody profiles

**Figure 3:**
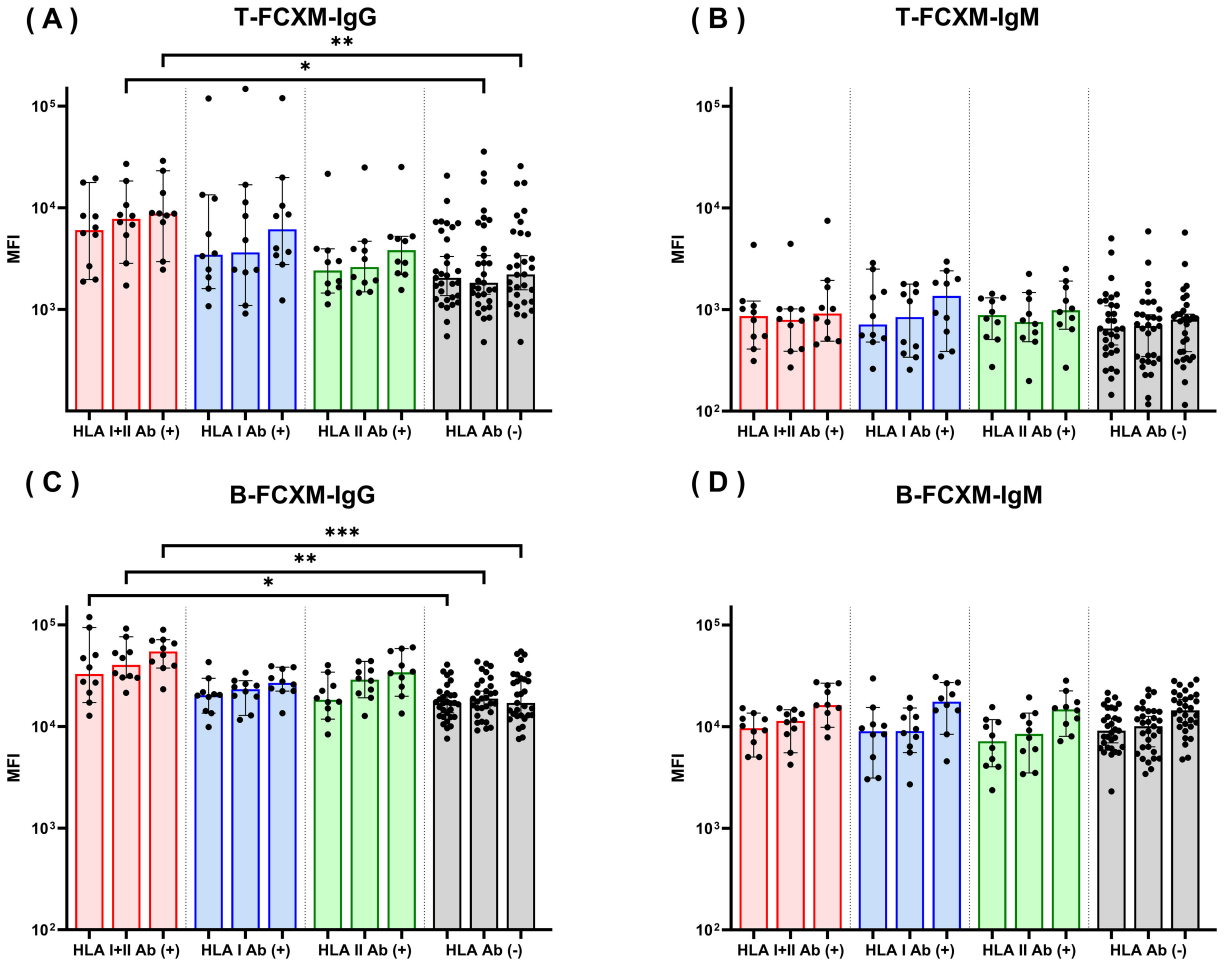
IgG and IgM reactivity of human sera against QKO pig PBMCs by HLA antibody profile. Flow cytometric crossmatch (FCXM) assays were performed with 61 human sera, categorized by HLA antibody status, against PBMCs from three QKO pigs (#1, #2, and #3; colored consistently left to right). Sera were stratified into four groups; HLA I+II Ab (+), HLA I Ab (+), HLA II Ab (+), and HLA Ab (–). IgG and IgM reactivities were measured for T-cell **(A, B)** and B-cell **(C, D)** subsets: T-FCXM-IgG **(A)**, T-FCXM-IgM **(B)**, B-FCXM-IgG **(C)**, and B-FCXM-IgM **(D)**. Sera positive for both HLA class I and II antibodies demonstrated significantly higher IgG binding to QKO pig T and B cells compared to HLA Ab **(-)** sera. In contrast, IgM binding did not differ significantly among groups. *p < 0.05, **p < 0.01, ***p < 0.001.

To assess the impact of HLA antibody status on antibody reactivity toward QKO pigs, FCXM assays were performed on both T and B cell subsets using 61 human sera samples and PBMCs from three individual QKO pigs (**Table 1**). Four serum groups were categorized according to their HLA antibody profiles. For IgG reactivity to T cells, sera positive for both HLA class I and II antibodies demonstrated significantly higher IgG binding compared to HLA antibody-negative sera in two of the three QKO pigs (QKO #2, #3), (p < 0.05), with a similar, but non-significant trend observed for QKO #1 (p = 0.15) (**Figure 3A**). For IgG reactivity to B cells, significantly increased binding was observed across all three QKO pigs (p < 0.05) (**Figure 3C**). In contrast, IgM reactivity to T cells was low across all serum groups regardless of HLA antibody status (**Figure 3B**). Similarly, while B-cell IgM reactivity was lower overall than IgG, MFI values did not differ significantly between any of the HLA antibody groups (**Figure 3D**). These results indicate that human sera broadly sensitized to HLA class I and II antigens have elevated IgG cross-reactivity with QKO pig PBMCs. Conversely, IgM responses remain consistently low irrespective of HLA sensitization status, suggesting that HLA sensitization predominantly associates with increased IgG—but not IgM—cross-reactivity toward xenogeneic targets.

**Table 1:** SLA typing of QKO pigs.

Pig ID	SLA haplotype	SLA class I			SLA class II		
		SLA-1*	SLA-2*	SLA-3*	DRB1*	DQA*	DQB1*
QKO #1	4.5	*04:01:01*	*04:02:01*	*04:01:01*	*05:01*	*02:02:02*	*02:01*
QKO #2	4.5	*04:01:01*	*04:02:01*	*04:01:01*	*05:01*	*02:02:02*	*02:01*
	6.7	*08:05*	*05:04*	*06:01*	*06:01*	*01:06*	*06:01*
QKO #3	4.5	*04:01:01*	*04:02:01*	*04:01:01*	*05:01*	*02:02:02*	*02:01*
	6.7	*08:05*	*05:04*	*06:01*	*06:01*	*01:06*	*06:01*

Each row corresponds to one SLA haplotype type for the indicated pig; the columns list alleles for class I loci (SLA-1, SLA-2, SLA-3) and class II loci (DRB1, DQA, DQB1). Each allele is designated according to the IPD-MHC nomenclature.

SLA, swine leukocyte antigen; QKO, quadruple knockout.

### 3.4 Cytotoxicity of human sera to QKO pig PBMCs according to the HLA antibody profiles

**Figure 4:**
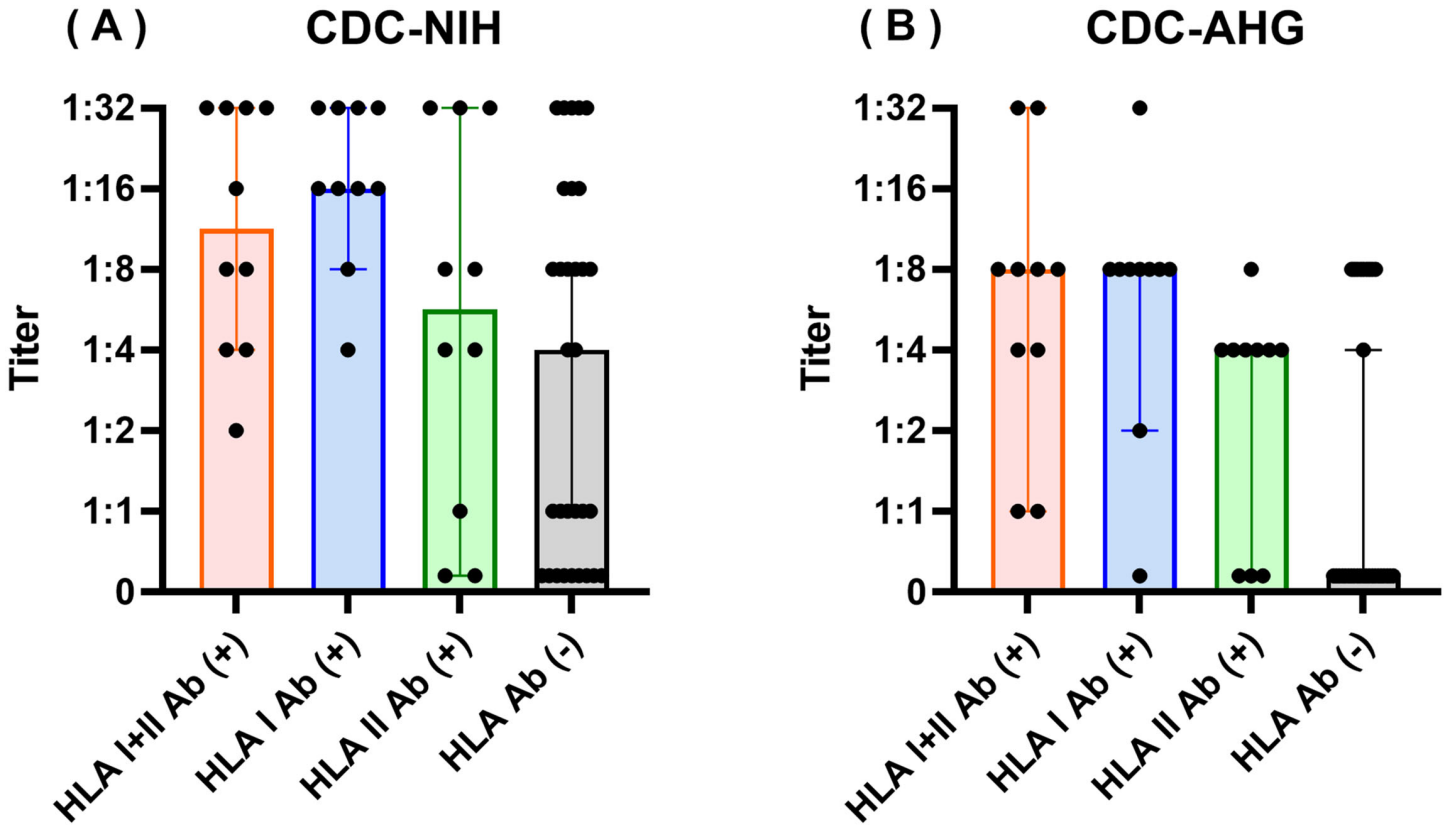
Complement-dependent cytotoxicity titers of human sera against QKO pig PBMCs by HLA antibody profile. Complement-dependent Cytotoxicity (CDC) assays were performed with 61 human sera against QKO #2 pig PBMCs using CDC-NIH **(A)** and CDC-AHG **(B)** assays. Sera were grouped by HLA antibody status; HLA I+II Ab (+), HLA I Ab (+), HLA II Ab (+), HLA Ab (–). Each dot indicates the CDC titer for an individual serum, defined as the lowest dilution achieving ≥40% cytotoxicity. HLA antibody-positive sera tended to exhibit higher cytotoxicity compared to HLA Ab **(-)** sera.

To evaluate the effect of HLA antibody status on cytotoxic responses against QKO pig PBMCs, CDC assays were performed with 61 human sera and PBMCs from QKO #2. In the CDC-NIH test, 9 of 10 HLA class I antibody-positive sera and 7 of 10 sera positive for both HLA class I and II antibodies exhibited CDC positivity with titers ≥1:8. In contrast, 16 of 31 HLA antibody-negative sera achieved titer ≥1:8 (**Figure 4A**). Similarly, the CDC-AHG assay—utilizing anti-human globulin to enhance sensitivity—demonstrated that 8 of 10 sera positive for HLA class I antibodies and 6 of 10 sera positive for HLA class I and II antibodies exhibited titers ≥1:8. The HLA antibody-negative group showed lower titers in the CDC-AHG compared to CDC-NIH. Among the antibody-negative sera, 10 of 31 samples were CDC-positive against QKO pig PBMCs at titers ≥1:4 (**Figure 4B**). Overall, CDC-AHG results demonstrated a higher frequency of cytotoxicity positivity in HLA antibody–positive sera compared to antibody–negative sera, consistent with the FCXM assay findings (**Supplementary Figure 2**).

### 3.5 HLA antibody and epitope analysis following elution from QKO pig PBMCs in allosensitized human sera

**Figure 5:**
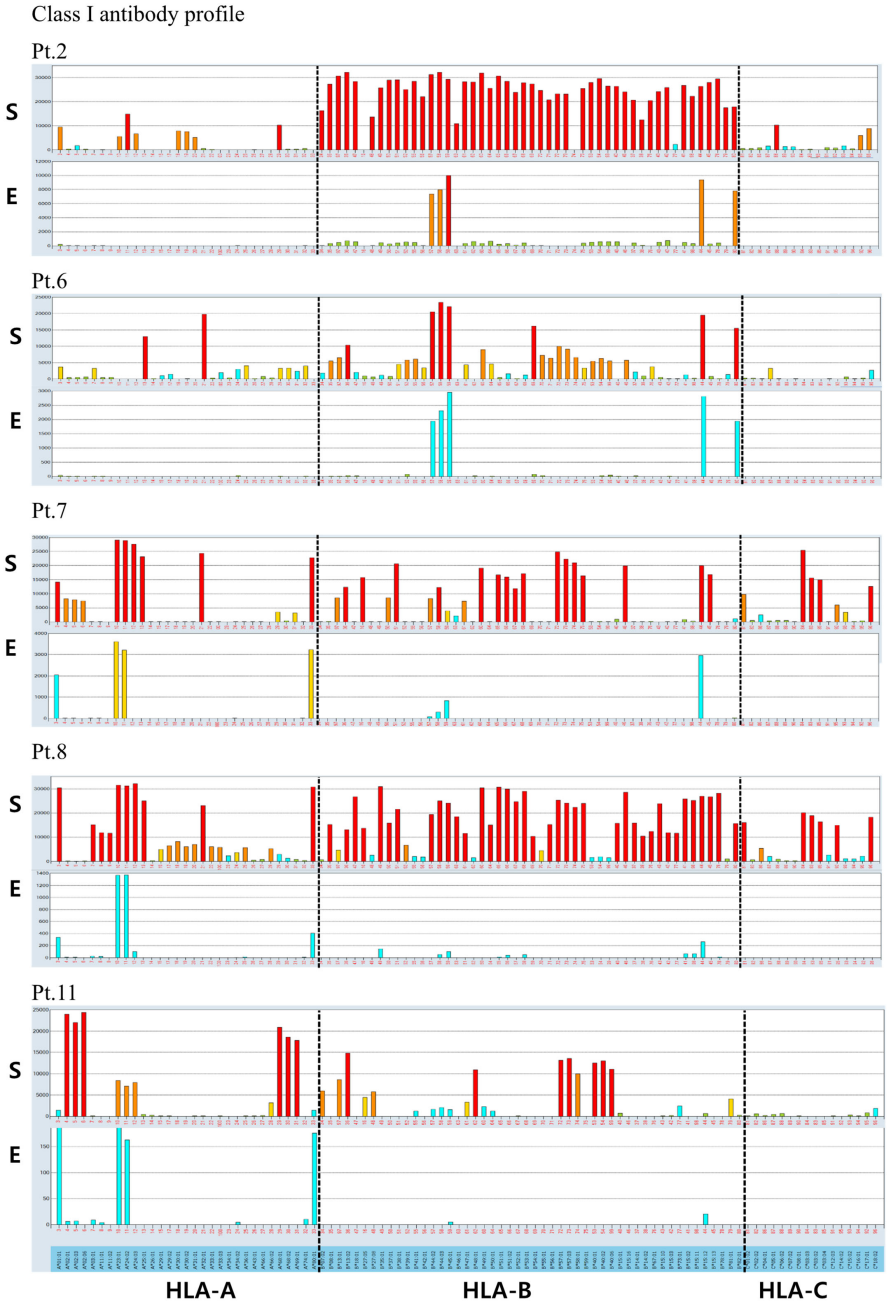
Antibody specificity profiles before and after elution from porcine PBMCs in allosensitized human sera assessed by HLA single antigen bead assay. Class I antibody reactivity profiles from five allosensitized human sera were compared before (serum, S; upper panels) and after (elute, E; lower panels) elution from QKO pig PBMCs using HLA single antigen bead assays. Class I antibody binding demonstrates that antibodies targeting HLA-A and HLA-B are predominant in both original sera and eluates, while HLA-C reactivity is absent. Bar colors indicate antibody strength (red: strong; yellow: moderate; cyan: weak).

**Figure 6:**
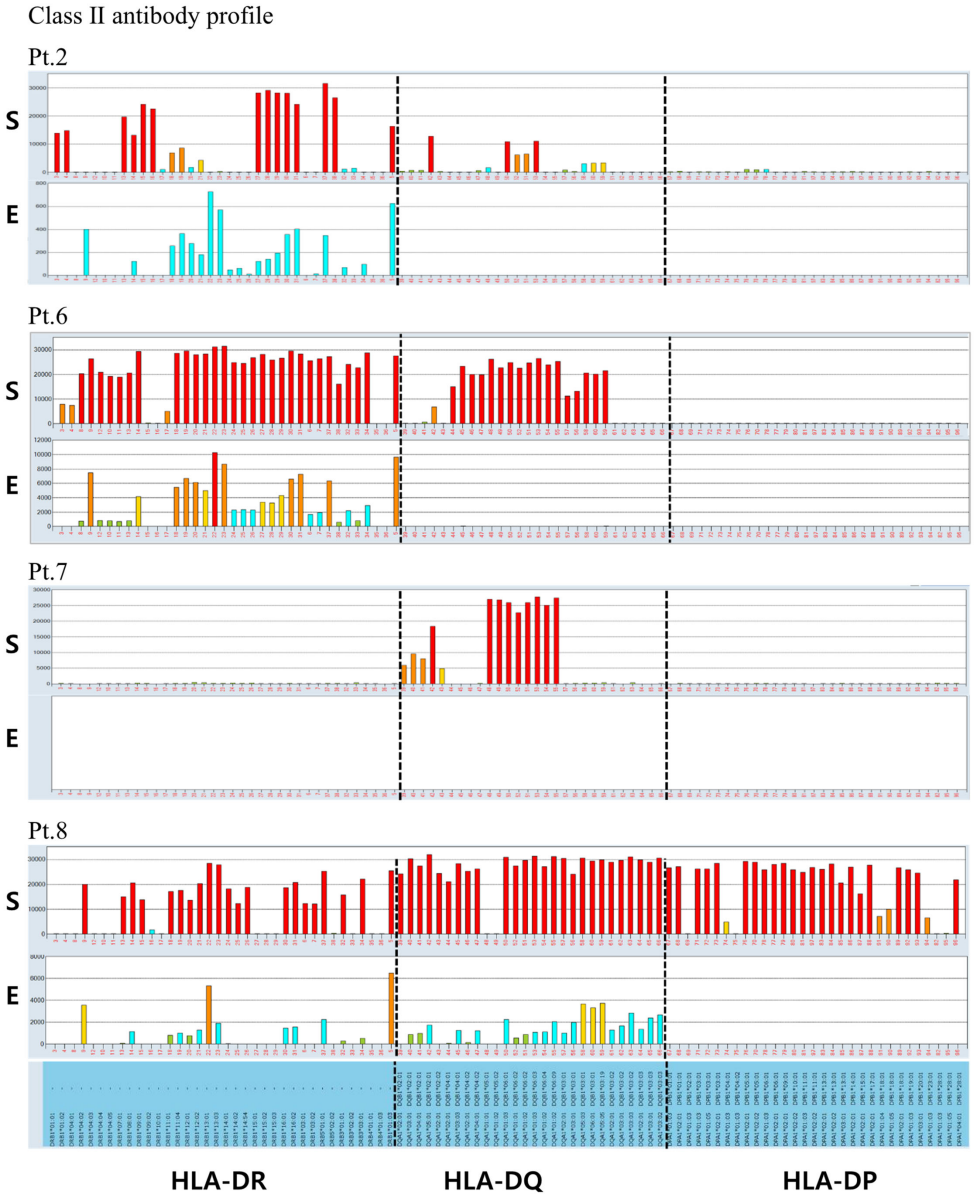
Class II antibody specificity profiles before and after elution from porcine PBMCs in allosensitized human sera assessed by HLA single antigen bead assay. Class II antibody reactivity profiles from five allosensitized human sera were compared before (serum, S; upper panels) and after (elute, E; lower panels) elution from QKO pig PBMCs using HLA single antigen bead assays. Class II antibody binding reveals that anti-HLA-DR antibodies are consistently detected after elution (3 of 3 samples), while anti-HLA-DQ antibodies are infrequently found (1 of 4 samples) and anti-HLA-DP responses are absent. Cross-reactive antibodies in eluates were largely confined to those with moderate or strong reactivity in the original sera (MFI >5,000). Bar colors indicate antibody strength (red: strong; yellow: moderate; cyan: weak).

To further elucidate antibody cross-reactivity between allosensitized human sera and SLA antigens on QKO pig PBMCs, antibody elution was performed on five human sera (#2, 6, 7, 8, 11) that had previously demonstrated crossmatch positivity against QKO #2 pig PBMCs. The HLA antibody and corresponding epitope profiles of the eluted fractions are summarized in **Table 2**. All five sera, each had strong HLA class I antibodies, demonstrated cross-reactive antibody binding to QKO pig PBMCs. Analysis of eluted class I antibody profiles demonstrated that those targeting HLA-A and HLA-B were predominant, with cross-reactive antibodies (highlighted in red in **Table 2**) mostly restricted to specificities with moderate to high reactivity (MFI >5,000). The C1q binding results for sample #2 were evaluated for complement activation (**Table 2**). Among the red-highlighted eluted antibodies, all class I except A1 and class II antibodies against DR51, DR15, and DR16 showed C1q positivity, indicating cross-reactive antibody complement activation. Identified class I cross-reactive epitopes included 62EE, 162GLS, 163LG, 163LS/G, 166ES, and 199V, implicating these epitopes in crossmatch-positive reactions in this setting. Of the four sera with strong HLA class II antibodies, three sera showed persistent antibody reactivity in the eluted fractions. Most class II reactivity was directed against HLA-DR epitopes, with consistency observed for 13SE, 37F, 47F, 70D, and 70DA across multiple samples. Despite strong anti–HLA-DQ antibody responses in native sera, only one eluted sample exhibited weak DQ-specific antibody directed against the 160D epitope. Importantly, cross-reactivity was not uniformly observed for all strong HLA antibodies. Broad, high-titer HLA antibodies responses did not necessarily result in cross-reactivity; rather, only certain epitope-specific antibodies with moderate to high reactivity (MFI >5,000) consistently showed cross-reactivity across multiple samples (**Figures 5**, **6**).

**Table 2:** Cross-reactive HLA eptiopes identified by antibody elution and single antigen bead assay in allosensitized human sera (n = 5).

Patient no	HLA class I Ab profile in human sera and eluted fraction (eluted Ab in red)	Potential cross-reactive class I epitope	HLA class II Ab profile in human sera and eluted fraction (eluted Ab in red)	Potential cross-reactive class II epitope
2	- Strong: B49, **B44**, **B13**, **B51**, **B78**, **B45**, **B38**, **B37**, **B41**, **B18**, **B47**, **B48**, **B61**, **B77**, **B60**, **B53**, B54, **B8**, **B62**, **B76**, **B72**, **B35**, **B50**, **B59**, **B39**, B55, **B75**, **B71**, B63, **B52**, B57, B42, B56, **B64**, B67, **B82**, B81, B7, B27, B65, B46, A24, Cw6, A68 - Moderate: **A1**, Cw18, A30, Cw17, A23, A31 - Weak-Moderate: - - Weak: B73, A2, Cw15, Cw5, Cw7, Cw8	**163LS/G** **166ES** **162GLS** **199V** **163LG**	- Strong: **DR51**, **DR15**, **DR16**, DR9, DR7, **DR103**, DR1, **DR8**, DQA1*05:01, DQA1*01:03, DQ6 - Moderate: **DR11**, DQA1*01:01 - Weak-Moderate: DQA1*05:05, DQA1*06:01, **DR12** - Weak: DQA1*05:03, DR52, DP6, DR10	**70DA** **70D** **47F**
6	- Strong: **B45**, **B44**, A32, **B76**, B54, **B82**, A25, B13 - Moderate: B57, B49, B55, B58, B56, B41, B61, B39, B63, B8, B60 - Weak-Moderate: B50, B38, B47, A36, A74, B67, A1, B42, B59, A3, A68, Cw5 - Weak: A34, Cw18, A69, B64, B18, A33, B7, B51, B81, B75, B53, A29, B35	**163LS/G** **166ES**) **162GLS** **199V** **163LG**	- Strong: **DR13**, **DR8**, **DR11**, **DR16**, **DR12**, **DR103**, **DR15**, **DR18**, DQA1*01:03, **DR17**, **DR14**, **DR52**, DQ6, DQ5, DQA1*01:01, DQA1*01:02, **DR51**, DQA1*05:05, **DR4**, **DR7**, DQA1*05:03, DQA1*06:01, DQ4, DQ7 - Moderate: DR1, DQA1*05:01, DR10 - Weak-Moderate: - - Weak: -	**70DA** **70D** **13SE** **37F**
7	- Strong: **A23**, **A24**, Cw9, A32, B57, A25, **A80**, B58, B38, **B76**, B63, B49, B53, B77, B59, B51, B27, Cw10, **A1**, Cw18, B52, B13, **B44** - Moderate: Cw1, B37, A2, B47, Cw14 - Weak-Moderate: **B45**, A68, Cw15, A69 - Weak: Cw4, B46, B82	**163LS/G** **166ES** **162GLS** **199V**	- Strong: DQ5, DQA1*01:03, DQA1*01:02, DQ6, DQA1*01:01, DQA1*05:01, DQ2 - Moderate: - - Weak-Moderate: - - Weak: -	
8	- Strong: **A24**, **A23**, **B35**, **A80**, B49, **A1**, B51, B53, B63, B78, **B76**, B77, B18, B75, A25, B52, B57, B45, B59, B71, A32, B58, B44, B38, Cw9, B46, Cw18, Cw10, Cw1, B37, B64, B62, B82, B8, B56, A3, B50, Cw14, B67, B72, A11, B73, B47, B65, B54, B13, B27 - Moderate: A30, A31, B39, A33, A29, A36, Cw4, A66 - Weak-Moderate: B55, A34 - Weak: Cw12, Cw17, A68, Cw5, B41, B42, B61, B60, B48, Cw15, Cw16	**62EE** **163LG**	- Strong: DQA1*05:01, DQA1*01:03, DQA1*05:03, DQA1*03:02, **DQ8**, DQA1*05:05, **DQ9**, DQA1*03:03, **DQ6**, DQA1*06:01, DQA1*01:02, DP5, **DQ7**, DQA1*03:01, **DR13**, **DQ2**, DPA1*02:02, DP3, DP1, DP6, DQA1*04:01, DPA1*02:01, DQA1*02:01, DP11, **DR103**, **DR51**, **DQ4**, DP13, DPA1*03:01, DPA1*04:01, **DR8**, DPA1*01:03, **DR4**, **DR16**, **DR52**, DPA1*01:05, **DR11**, **DR12**, DR14, DR7, DP18, DR17, DR18, DR9 - Moderate: DPA1*01:04 - Weak-Moderate: - - Weak: -	**70DA** **160D**
11	- Strong: A2, A68, A69, B57, B60, B61, B13, B48 - Moderate: B58, **A23**, **A24**, B7, B27 - Weak-Moderate: B81, B47, A66 - Weak: B73, B49, Cw18, B44, B45, **A1**, **A80**, B50, B41	**62EE**	- Strong: - - Moderate: - - Weak-Moderate: - - Weak: DQ4	

HLA antibody profiles in five sera and eluted fraction are shown, with eluted antibodies highlighted in red. Potential cross-reactive eptiopes identified in the eluted fraction are indicated in bold.

### 3.6 In silico analysis of eluted HLA epitopes within SLA sequences and on structural surfaces

To confirm the sequence conservation of eluted HLA epitopes in SLA proteins, SLA sequences were aligned with HLA reference sequences using NCBI BLASTP. Multiple HLA eplets identified in eluates were positionally conserved in the homologous regions of corresponding SLA alleles (**Table 3**). For class I eplets, 62EE was found in SLA-2*05:04 and SLA-3*06:01; 162GLS and 166ES in SLA-1*04:01; 163LG in SLA-1*08:05 and SLA-2*05:04; 163LS/G in SLA-1*04:01, SLA-1*08:05, and SLA-2*05:04; and 199V in SLA-1*04:01, SLA-1*08:05, SLA-2*04:02:01, SLA-3*04:01, and SLA-3*06:01. For class II eplets, 13SE in SLA-DRB1*06:01; 37F in SLA-DRB1*05:01 and SLA-DRB1*06:01; 47F, 70D, and 70DA were found in SLA-DRB1*05:01; and 160D in SLA-DQA*02:02:02 and SLA-DQA*01:06.

**Table 3:** Shared and cross-reactive eplets between human HLA and porcine SLA molecules.

HLA class I eplet	Associated HLA class I alleles	Associated SLA class I alleles in this study	HLA class II eplet	Associated HLA class II alleles	Associated SLA class II alleles in this study
62EE	A*23:01, A*24:02, A*24:03, A*80:01	SLA-2*05:04, SLA-3*06:01	13SE	DRB1*03:01, DRB1*03:02, DRB1*03:03, DRB1*11:01, DRB1*11:03, DRB1*11:04, DRB1*13:01, DRB1*13:02, DRB1*13:03, DRB1*13:05, DRB1*14:01, DRB1*14:02, DRB1*14:03, DRB1*14:05, DRB1*14:06, DRB1*14:54, DRB3*01:01, DRB3*02:01, DRB3*02:02, DRB3*03:01	SLA-DRB1*06:01
162GLS	B*44:02, B*44:03, B*45:01, B*50:02, B*82:02	SLA-1*04:01	37F	DRB1*07:01, DRB1*14:01, DRB1*14:04, DRB1*14:05, DRB1*14:54, DRB3*01:01, DRB3*03:01	SLA-DRB1*05:01, SLA-DRB1*06:01
163LG	B*15:12	SLA-1*08:05, SLA-2*05:04	47F	DRB1*03:01, DRB1*11:01, DRB1*11:03, DRB1*11:04, DRB1*12:01, DRB1*12:02, DRB1*13:01, DRB1*13:02, DRB1*13:05, DRB1*15:01, DRB1*15:02, DRB1*15:03	SLA-DRB1*05:01
163LS/G	B*15:12, B*44:02, B*44:03, B*45:01, B*50:02, B*82:01, B*82:02	SLA-1*04:01, SLA-1*08:05, SLA-2*05:04	70D	DRB1*01:03, DRB1*04:02, DRB1*07:01, DRB1*08:01, DRB1*08:02, DRB1*08:03, DRB1*08:07, DRB1*11:01, DRB1*11:03, DRB1*11:04, DRB1*12:01, DRB1*12:02, DRB1*13:01, DRB1*13:02, DRB1*13:03, DRB1*13:05, DRB1*14:03, DRB1*16:01, DRB1*16:02, DRB5*01:01, DRB5*01:02	SLA-DRB1*05:01
166ES	B*44:02, B*44:03, B*45:01, B*50:02, B*82:01, B*82:02	SLA-1*04:01	70DA	DRB1*01:03, DRB1*04:02, DRB1*08:01, DRB1*08:02, DRB1*08:03, DRB1*08:07, DRB1*11:01, DRB1*11:03, DRB1*11:04, DRB1*12:01, DRB1*12:02, DRB1*13:01, DRB1*13:02, DRB1*13:03, DRB1*13:05, DRB1*14:03, DRB1*16:01, DRB1*16:02, DRB5*01:01, DRB5*01:02	SLA-DRB1*05:01
199V	B*44:02, B*44:03	SLA-1*04:01, SLA-1*08:05, SLA-2*04:02:01, SLA-3*04:01, SLA-3*06:01	160D	DQA1*03:02, DQA1*03:03	SLA-DQA*02:02:02, SLA-DQA*01:06

**Figure 7:**
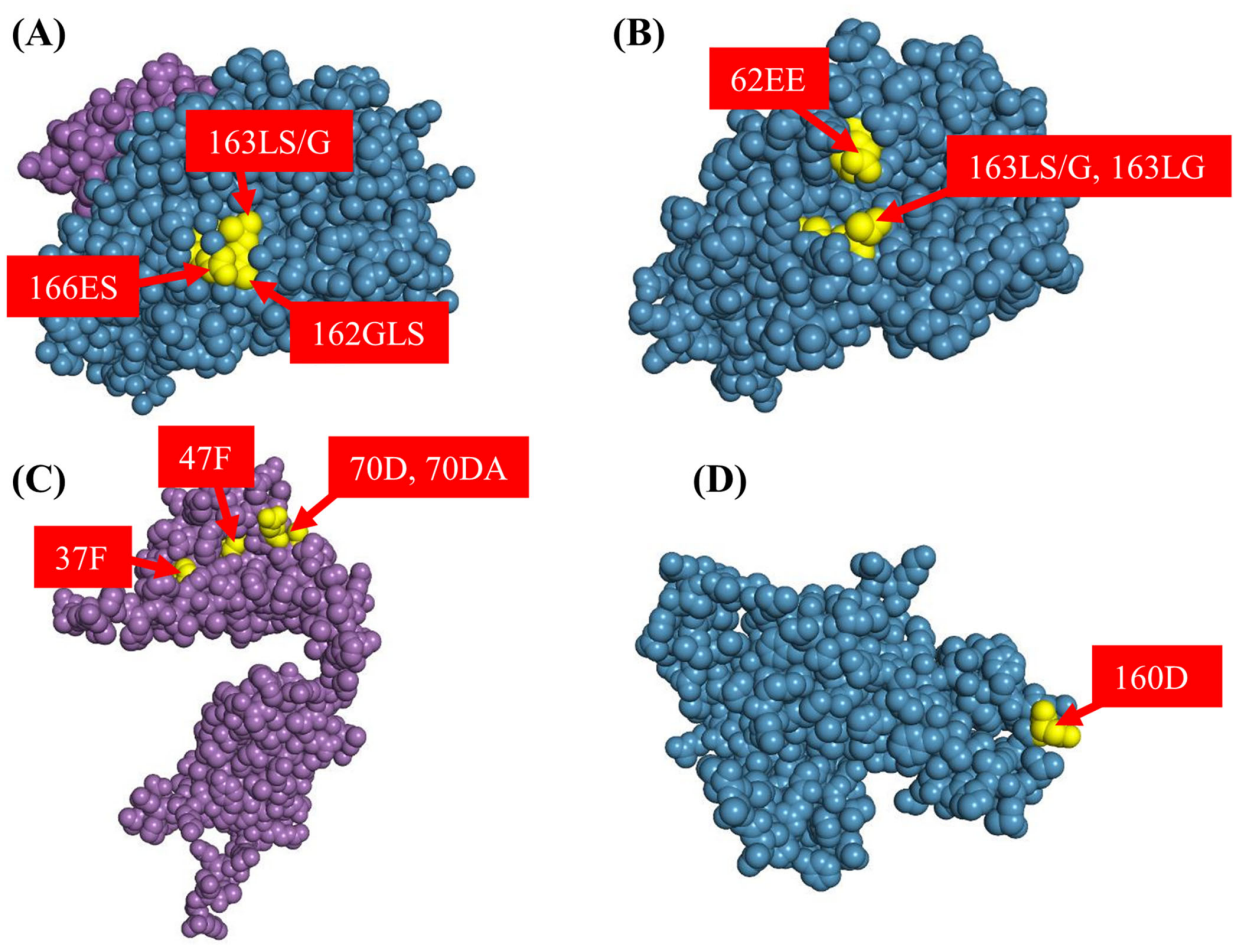
In silico structural analysis of eluted antibody eplets on porcine SLA molecules. Target eplets identified by antibody elution were mapped onto the surfaces of SLA proteins; **(A)** SLA-1*04:01, **(B)** SLA-2*05:04, **(C)** SLA-DRB1*05:01, **(D)** SLA-DQA*02:02:02. SLA structure were derived from the RCSB Protein Data Bank, AlphaFold-predicted models, and the ESMfold model. The α-chain is shown in purple, and the β-chain in blue, and eplet residues in yellow.

Next, we evaluated whether these eplets were surface-exposed and appeared potentially accessible for antibody binding in the SLA molecules of QKO #2. Structural accessibility was analyzed using PyMOL, based on reference structures from the Protein Data Bank (PDB), AlphaFold-predicted models, and the ESMFold model. All identified eplets were confirmed to be surface-exposed and thus accessible for antibody binding (**Figure 7**).

## 4 Discussion

As xenotransplantation approaches clinical trials, it is critical to confirm cross-reactivity and accurately assess immunologic risk, especially for highly sensitized patients who are the most appropriate candidates. This study provides comprehensive insights into cross-reactive HLA antibody responses against genetically modified pig cells, specifically from QKO pigs.

We first evaluated baseline xenoreactive antibody responses in the absence of anti-HLA sensitization. Normal human sera contained significant levels of natural xenoreactive IgG and IgM targeting WT and GTKO porcine PBMCs, with a substantial reduction in antibody binding observed in GGTA1/CMAH/B4GALNT2 triple knock-out pigs, including TKO, QKO, and TKO pigs expressing human protective transgenes. This confirms that the majority of natural human xenoantibody reactivity is directed against carbohydrate antigens absent in these gene-edited pigs (1, 28).

Subsequently, we assessed the necessity of RBC adsorption in immunologic assays with QKO pigs. The marked reduction in IgG and IgM binding to WT pig PBMCs following RBC adsorption is consistent with previous report demonstrating that baseline xenoreactive humoral responses are largely driven by natural antibodies against carbohydrate epitopes such as α-Gal, Neu5Gc, and Sda present on porcine cells (28). In contrast, RBC adsorption did not further reduce IgG or IgM binding to QKO PBMCs, which lack all major glycan xenoantigens, indicating that RBC adsorption is unnecessary when using PBMCs from QKO donors in crossmatch testing.

When analyzing antibody reactivities to QKO pigs in sera from patients with HLA antibodies, we found that human sera broadly sensitized to both HLA class I and class II antigens exhibited significantly increased IgG cross-reactivity to QKO pig PBMCs in both T- and B-cell subsets. This suggests that anti-HLA sensitization poses a barrier not only to allotransplantation but also to xenotransplantation by promoting antibody binding to SLA and other porcine targets, even in gene-edited donors. The increase in IgG—but not IgM—reactivity indicates involvement of memory B-cell or class-switched humoral responses, supporting evidence of shared or structurally similar epitopes between human HLA and porcine SLA (16–18, 29–31). The consistently low IgM responses across all groups suggest polyreactive natural antibodies are less likely to be responsible for cross-reactivity after removal of major glycan xenoantigens.

Our data also show that preformed HLA antibodies influence CDC assays against QKO pig PBMCs. In line with FCXM results, CDC assays, especially CDC-AHG, revealed that HLA class I–positive and class I/II double-positive sera exhibited increased cytotoxicity titer compared to HLA antibody–negative sera. Although CDC-NIH showed positivity in 51.6% of HLA antibody–negative sera at ≥1:8 dilution, CDC-AHG demonstrated lower overall titers yet preserved the pattern of significantly higher cytotoxicity in HLA antibody–positive sera. These findings suggest CDC-NIH positivity may be influenced by non-HLA antibodies or other cross-reactive factors in addition to anti-HLA/SLA cross-reactivity. The difference in CDC titers between CDC-NIH and CDC-AHG assays reflects the complexity of accurately assessing humoral risk and the need for sensitive, reliable assays in the xenotransplant setting.

Our most novel contribution lies in elucidating epitope-level specificity of cross-reactive antibodies. The detection of cross-reactive antibody binding in sera with strong HLA class I reactivity reveals that certain HLA alloantibodies can recognize shared or structurally similar epitopes on porcine SLA. Identified class I eplets (62EE, 162GLS, 163LG, 163LS/G, 166ES, and 199V) targeting SLA 4.5/6.7 haplotypes imply that molecular mimicry or epitope conservation underlies these crossmatch-positive reactions. This finding is concordant with recent studies employing eplets and sequence homology analyses to map HLA-SLA cross-reactivity in xenotransplantation candidates (30, 32). Similarly, persistent antibody reactivity in strong HLA class II sensitized sera mainly targeted HLA-DR–associated epitopes (13SE, 37F, 47F, 70D, and 70DA). The consistent presence of anti-DR epitope antibodies across multiple individuals points to DR-based epitopes as key mediators of class II cross-reactivity. Despite strong anti–HLA-DQ responses before elution, DQ-specific antibodies bound weakly and infrequently to QKO pig PBMCs expressing SLA 4.5/6.7 haplotype. This aligns with previous report indicating that structural and sequence divergence between human HLA-DQ and porcine SLA-DQ restricts effective cross-species antibody binding (18).

Importantly, not all broadly reactive or high-titer HLA antibodies cause cross-reactivity. Only antibodies with moderate to high MFI (>5,000) directed against specific epitopes consistently demonstrated cross-reactivity, emphasizing antibody specificity and epitope structures as critical determinants of binding to pig SLA antigens. In addition, C1q binding of cross-reactive antibodies (sample #2) demonstrates their ability to activate complement, which is linked to higher antibody-mediated rejection risk and poorer graft outcomes. These results expand understanding of xenotransplant humoral immunity and support integrating high resolution epitope mapping alongside traditional antibody profiling. Such detailed analyses can improve compatibility assessments and guide personalized immunomodulatory or genetic engineering strategies to reduce antibody-mediated xenograft rejection risk.

This study has limitations, including a restricted number and diversity of pig SLA haplotypes, limiting generalizability. Expanding alleles coverage in future work will clarify prevalence and impact of cross-reactive epitopes. The use of PBMCs instead of endothelial or graft-derived cells in this study may not accurately reflect xenograft antigen profiles, and the limited number of highly sensitized human sera further constrains broader interpretation. Future study will require expanded sample sets and established porcine endothelial cell lines to validate our results. Cross-reactivity could also be influenced by non-HLA antibodies or other factors not addressed here. In addition, the pig strain used in this study for elution assays was limited to QKO pigs with a defined set of SLA haplotypes. Given the increasing variety of genetically engineered pig lines worldwide, each with diverse SLA haplotypes, the generalizability of our findings is constrained. Expanding the allelic diversity in future studies will enhance understanding of cross-reactive epitope prevalence and impact. Second, although adsorption with QKO pig red blood cells substantially reduced the confounding effects of xenogeneic glycan antibodies, potential interference by non-SLA porcine protein antigens remains possible. Additional cellular and functional assays are needed to determine whether the identified cross-reactive SLA epitopes directly mediate immune responses or represent secondary binding phenomena. Structural analysis relied on limited experimentally determined crystal structures supplemented by AI-predicted models. Additionally, assay sensitivity across discrepancies needs assay standardization to enhance comparability and inform clinical decisions reliably.

In conclusion, this study reveals that cross-reactive anti-SLA antibodies are common in allosensitized human sera and that their binding profiles are shaped by the specificity, strength, and epitope-targeting characteristics of anti-HLA responses. While elimination of key carbohydrate xenoantigens in engineered pigs reduces natural antibody binding, sensitized human sera with anti-HLA antibodies still show increased IgG-mediated cross-reactivity and cytotoxicity against pig cells. These findings emphasize the need for detailed antibody and epitope profiling to refine recipient selection and improve outcomes in clinical xenotransplantation. Future studies should broaden allele representation and validate epitope predictions to optimize risk assessment and immunomodulation strategies.

## Data Availability

The original contributions presented in the study are included in the article/**Supplementary Material**. Further inquiries can be directed to the corresponding author.

## Supplementary material

Supplementary Figure 1 Flowcytometric analysis of IgG and IgM binding to WT and QKO pig PBMCs before and after the RBC adsorption. Black histograms indicate antibody binding after RBC adsorption. White histograms represent binding before adsorption. RBC adsorption markedly reduced IgG and IgM reactivity to WT PBMCs in both T and B cells, while no significant changes were observed in QKO PBMCs.

Supplementary Figure 2 T- and B-cell FCXM-IgG and CDC-AHG assay results of 61 human sera against QKO pig PBMCs by HLA antibody profile. Sera were grouped by HLA antibody status; HLA I+II Ab (+), HLA I Ab (+), HLA II Ab (+), HLA Ab (–). Individual serum responses are shown for **(A)** T-cell FCXM-IgG reactivity, **(B)** B-cell FCXM-IgG reactivity, and **(C)** CDC-AHG titers. The horizontal dashed lines indicate the median value of the HLA I+II Ab (+) group. Sera 2*, 6*, 7*, 8*, and 11* were selected for single antigen bead analysis after elution.
