# Supplementary figures and images for: Epitope-level analysis of cross-reactive human HLA antibodies against genetically modified swine leukocyte antigens in xenotransplantation
Source: Front Immunol. 2025 Dec 15;16:1712793. doi: 10.3389/fimmu.2025.1712793 (PMC12745215; doi:10.3389/fimmu.2025.1712793)

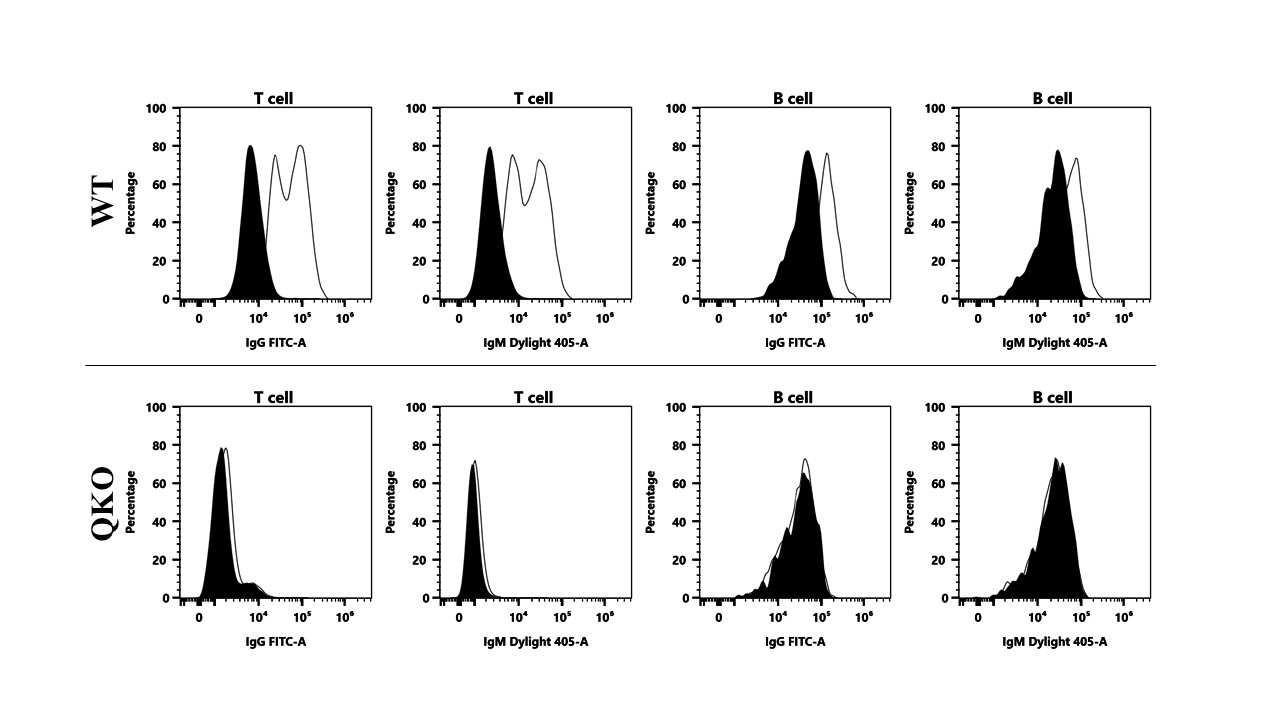

Supplement: Supplementary Figure 1 — Flowcytometric analysis of IgG and IgM binding to WT and QKO pig PBMCs before and after the RBC adsorption. Black histograms indicate antibody binding after RBC adsorption. White histograms represent binding before adsorption. RBC adsorption markedly reduced IgG and IgM reactivity to WT PBMCs in both T and B cells, while no significant changes were observed in QKO PBMCs. [file Image1.jpeg]

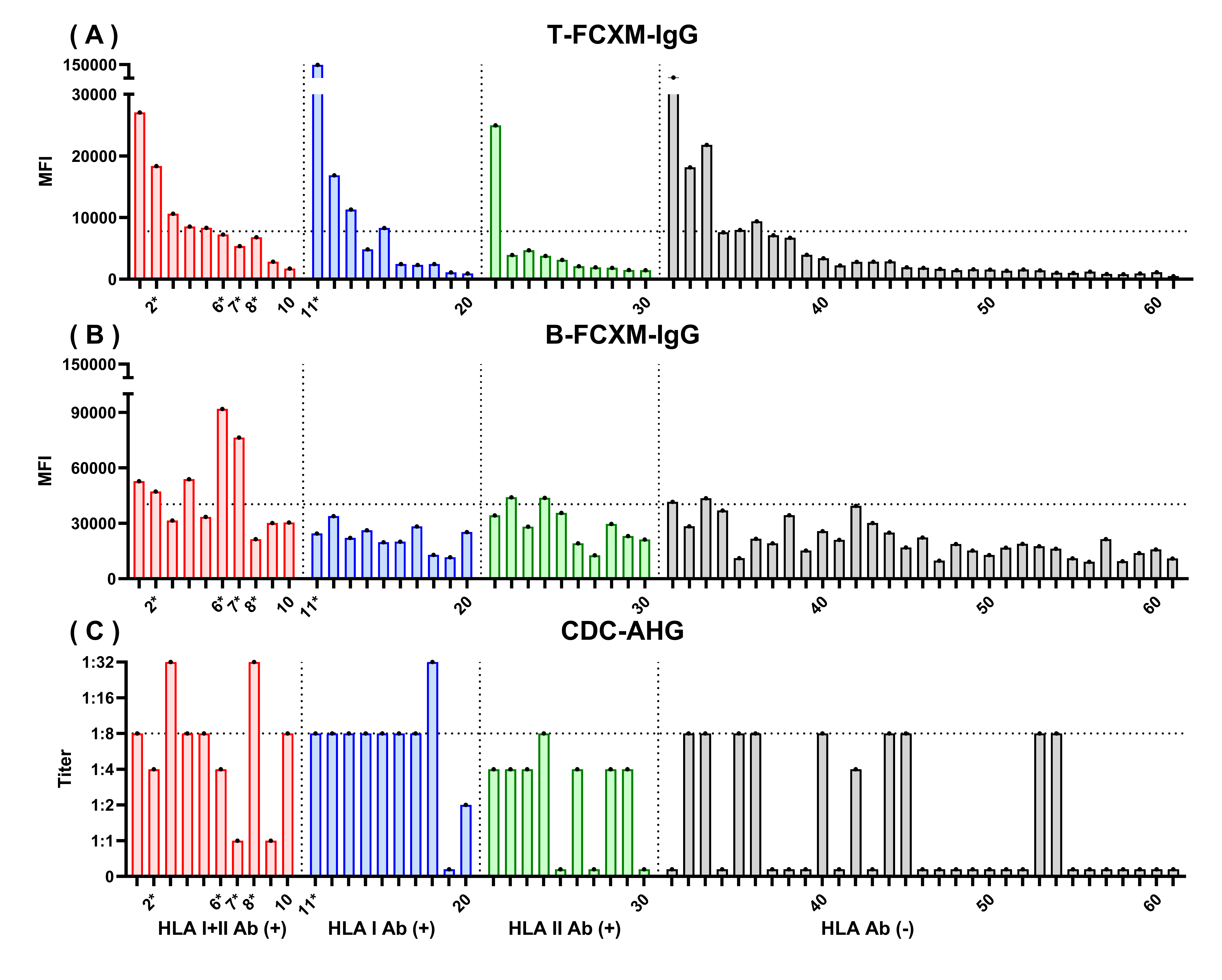

Supplement: Supplementary Figure 2 — T- and B-cell FCXM-IgG and CDC-AHG assay results of 61 human sera against QKO pig PBMCs by HLA antibody profile. Sera were grouped by HLA antibody status; HLA I+II Ab (+), HLA I Ab (+), HLA II Ab (+), HLA Ab (–). Individual serum responses are shown for (A) T-cell FCXM-IgG reactivity, (B) B-cell FCXM-IgG reactivity, and (C) CDC-AHG titers. The horizontal dashed lines indicate the median value of the HLA I+II Ab (+) group. Sera 2*, 6*, 7*, 8*, and 11* were selected for single antigen bead analysis after elution. [file Image2.tif]
